# Diagnostic and Treatment Challenges of Paroxysmal Nocturnal Hemoglobinuria in Uganda

**DOI:** 10.1155/2019/7897509

**Published:** 2019-01-31

**Authors:** Boniface Amanee Elias Lumori, Daniel Muyanja

**Affiliations:** Department of Internal Medicine, Mbarara University of Science and Technology (MUST), Mbarara, Uganda

## Abstract

**Introduction:**

Paroxysmal nocturnal hemoglobinuria (PNH) is a very rare disorder of the hematopoietic stem cells which is often underdiagnosed. Its incidence is about 5 cases per million inhabitants in a year, and currently, there are only 1610 patients in the International PNH Registry. In this report, we describe a case of PNH in southwestern Uganda.

**Case Presentation:**

A 34-year-old man, subsistence farmer, with a history of multiple prior presentations with anemia, jaundice, and dark-colored urine requiring blood transfusions presented to us again in July 2018 with a week's history of palpitations, dizziness, and dark-colored urine. Investigations done suggested a direct antiglobulin test- (DAT-) negative hemolytic anemia, and subsequently, flow cytometry showed a large clone of PNH. He received many blood transfusions and hematinics on several occasions during the course of his admissions.

**Conclusions:**

Our report showed diagnostic and treatment challenges of PNH in health resource-limited setting.

## 1. Introduction

Direct antiglobulin test-negative hemolytic anemia is a broad class of diseases characterized by hemolysis and a negative DAT. Paroxysmal nocturnal hemoglobinuria is one of the very rare causes of direct antiglobulin test-negative hemolytic anemia which is often underdiagnosed. It is an acquired hematopoietic stem cells disorder characterized by a variety of clinical features ranging from episodes of hemolysis and its associated symptoms, abdominal pain, erectile dysfunction, and thrombotic events to the renal insufficiency and pulmonary hypertension [1]. The reported incidence of PNH was only about 5 cases per million inhabitants per year [2]; however, it is associated with high morbidity and mortality [1]. Currently, there are only 1,610 patients with confirmed PNH in the International PNH Registry in which variable features of the disease are being studied [3]. To the best of our knowledge, there is no reported case of PNH confirmed by flow cytometry in Uganda. Therefore, in this report, we present a case of a 34-year-old man diagnosed with PNH by flow cytometry after 12 years a diagnostic dilemma.

## 2. Case Presentation

A 34-year-old man, a subsistence farmer, from southwestern Uganda with a history of multiple prior presentations with anemia, jaundice, and dark-colored urine requiring blood transfusions presented to us again in July 2018 with a week history of palpitations, dizziness, and dark-colored urine.

His condition started in 2006 with an episode of palpitations, yellowing of eyes, and dark-colored urine where he was initially seen in different health facilities close to his home village and later admitted to Mbarara Regional Referral Hospital (MRRH). He recalled being transfused with >4 units of blood during that initial admission and was discharged when all his symptoms subsided.

After discharge, he stayed fairly well for about 3 months before he developed another episode with similar symptoms. These symptoms continued to recur at an interval of 2–4 months, and each episode would require admission and blood transfusion.

In 2012, he was referred to Mulago National Referral Hospital for diagnostics and management. Many investigations were done (Table 1), and he was ultimately given a diagnosis of vitamin B12 deficiency. He was then treated for 1 year with vitamin B12 injections (no records of the doses available). Despite this treatment, he continued to have episodes of yellowing of eyes, palpitations, and dark-colored urine at approximately similar intervals (2–4 months).

In 2013, investigations were repeated, and in addition, bone marrow aspiration was done. The serum B12 level was found to be high, and the vitamin B12 injections were stopped. However, similar symptoms continued to recur at similar intervals over the following 2 years.

In 2015, he was restarted on B12 injections when found to have high serum levels of homocysteine despite a negative urine methylmalonic acid. The injections were stopped again a year later when found to have a very high serum B12 levels. Symptoms continued to recur at similar intervals till his recent admission in July 2018. Apart from B12 injections, the patient was given oral prednisolone on two occasions in the past but without significant improvement.

On this admission, he presented with predominant symptoms of palpitations, dizziness, generalized body weakness, yellow eyes, and dark-colored urine for about 7 days. This time, he also reported a 5-month history of erectile dysfunction and intermittent mild to moderate abdominal pain without associated vomiting, diarrhea, or dark/bloody stools. Reviews of the other systems were uneventful. He has no other chronic diseases or history of allergies. He has not been on any chronic medications in the past, apart from the tablets of folic acid and ferrous sulfate and vitamin B12 injections. He reported no history of a similar condition in any of his family members or a history of hereditary anemias or hematological malignancies. He reported no history of radiation or toxin exposure and further denied any history of taking traditional remedies.

His physical examination in the latest admission revealed severe pallor and jaundice of the mucous membranes. He had a displaced point of maximum cardiac impulse (6th left intercostal space and anterior axillary line) and grade-3 mitral and tricuspid murmurs of mitral and tricuspid regurgitation, respectively. He has no skin rashes, and the rest of his systemic examination was unremarkable. Many tests done during the course of his illness are displayed in Table 1.

Due to the recurrence of the symptoms, DAT-negative hemolytic anemia, and new onset of erectile dysfunction, we did a flow cytometry including fluorescent aerolysin (FLAER) in which a large PNH clone was found. The details of the flow cytometry test are displayed in Table 2.

In this admission, we transfused him with 4 units of blood and later discharged when his symptoms subsided.

## 3. Discussion

It took 12 years for our patient to be diagnosed with PNH, which is a little bit longer compared to the cases reported from Asia [1]. This is probably due to the lack of awareness of the disease and diagnostic capabilities in our setting and sub-Saharan Africa in general.

Our patient presents with a recurrence of hemolysis with a negative DAT, features which are suggestive of nonautoimmune hemolytic anemia. Causes of nonautoimmune hemolytic anemias include drugs, malaria, microangiopathic anemias, red blood cells enzymatic defects, and hereditary red blood cell membranes defects as well as paroxysmal cold hemoglobinuria and PNH. Unlike PNH, the hereditary anemias such as sickle cell anemias, thalassemia, hereditary spherocytosis, glucose-6-phosphate dehydrogenase (G6PD) deficiency, and pyruvate kinase have characteristic morphologies in the peripheral smear and positive family history [2]. In addition, these anemias do not have a glycosylphosphatidylinositol- (GPI-) anchored protein deficiency. Drugs/toxins exposure can also cause hemolysis but often stops when the insult is removed. Drugs/toxins exposure can typically manifest with recurrence if the patient is reexposed [3]; however, from our patient's history, there was no any exposure to drugs/toxins before the onset of his symptoms.

Malaria is common in Uganda, and it can cause anemia/hemolysis. Severe anemia is a frequent finding in complicated malaria caused by falciparum malaria. It is difficult to completely rule out malaria in our case, despite negative rapid malaria diagnostic test on some occasions of his admissions. Uganda is an endemic area for malaria; hence, it is unlikely to cause severe disease in adults due to the acquired partial immunity [4]. Blackwater fever is another manifestation of severe malaria characterized by hemolysis, hemoglobinuria, and renal failure [5]. These features are similar to those in our case; however, the recurrence pattern of his condition may rule out blackwater fever.

Disseminated intravascular coagulation, hemolytic uremic syndrome, and thrombotic thrombocytic purpura can similarly manifest with DAT-negative hemolysis; however, the absence of schistocytes on the peripheral smear in addition to the deficiency of GPI-anchored proteins in the flow cytometry ruled out these disorders. Few instances of autoimmune hemolytic anemias present with negative DAT similar to PNH and other DAT-negative hemolytic anemias. Most of the hemolysis in autoimmune hemolytic disorders are extravascular, and therefore, hemoglobinemia and hemoglobinuria are less evident [6].

The absence of any specific trigger for hemolysis in our patient may rule out paroxysmal cold hemoglobinuria which can be triggered by infections and cold temperature. Paroxysmal cold hemoglobinuria is also characterized by the presence of Donath–Landsteiner type antibodies but a negative flow cytometry [6].

Unsurprisingly, on several occasions, our patient was found with pancytopenia (Table 1) which is one of the common features reported in the International PNH Registry. These can be explained by the bone marrow suppression associated with this condition and/or lysis of all the blood cells lineage [7].

Erectile dysfunction and abdominal pain in our patient are likely from the smooth muscle dystonia caused by depletion of nitric oxide by the free circulating hemoglobin. In the PNH Registry, abdominal pain and erectile dysfunction were as common as 44% and 38%, respectively [7]. Our patient had intermittent abdominal pain; unfortunately, the abdominal vascular scan was not done to confirm the presence or absence of abdominal veins thrombosis, which is a serious complication in PNH patients. The normal abdominal scan may exclude the presence of biliary cholecystitis.

Our patient has dilated cardiomegaly which was similarly found in one of the cases of PNH in India [1]. The cardiomegaly in PNH can be attributed to recurrent anemia as well as iron deficiency caused by hemoglobinuria [8, 9].

Similar to the case found in India, our patient has features of megaloblastic anemia in peripheral smear and bone marrow aspirate in addition to the slightly low serum B12 levels. This might suggest the presence of low B12 status or B12 deficiency. Three years later, a negative urine methylmalonic acid but a high serum homocysteine level was found in our patient which seemed to suggest folate deficiency, despite the fact that both serum B12 and folate levels were not done at that time. Folate and B12 deficiencies are common findings in PNH mainly due to increased compensatory hematopoiesis secondary to chronic hemolysis [10].

Paroxysmal nocturnal hemoglobinuria is mostly a disease of adults, although childhood cases have been reported [11]. The median age of onset is in the thirties [12]. In Africa, we only found two published cases of PNH from South Africa about 6 decades ago [13]. Dr. Paul Strubing in 1882 described a young man who presented with fatigue, abdominal pain, and severe nocturnal paroxysms of hemoglobinuria [14]. In 1937, Ham reported that PNH erythrocytes were broken when incubated with normal, acidified serum [15], and this discovery resulted in the first diagnostic test for PNH, the acidified serum (Ham) test. In 1954, the alternative pathway of complement activation was formally proven to cause the hemolysis of PNH red cells. Later in the 1980s, it was discovered that PNH cells display a global deficiency in a group of proteins affixed to the cell surface by a GPI anchor and two of the missing GPI-anchored proteins (CD55 and CD59) regulate complement.

In PNH, hemolysis is complement mediated due to the PNH cells acquiring deficiency of GPI which are complement regulatory factors. The hematopoietic stem cells with severe deficiency of GPI on their surface progressively expand. The extent of this expansion determines the onset of the disease and its severity. The absence of GPI anchor in all PNH cases is caused by the somatic mutation in phosphatidylinositol glycan class A (PIGA), which is an x-linked gene required for the early step in GPI proteins synthesis. This leads to the deficiency of complement inhibitory proteins (CD55 and CD59) that result in chronic complement-mediated lysis of the GPI-deficient blood cells [7].

The International PNH Registry summarizes the clinical features of patients with PNH, and about 93% of 1610 patients in the registry are symptomatic with fatigue (80%), dyspnea (64%), and hemoglobinuria (62%) representing the most frequent features, then followed by abdominal pain (44%), bone marrow suppression (44%), and erectile dysfunction (38%). In addition, chest pain (33%), thrombosis (16%), and renal dysfunction (14%) are rarely reported [12]. Anemia in PNH is often caused by multiple factors with hemolysis and bone marrow failure as the major causes. Thrombosis in PNH occurs commonly in the intra-abdominal (hepatic, portal, mesenteric, splenic, etc) and cerebral (sagittal and cavernous sinus) veins.

The initial evaluation of a suspected patient includes CBC, reticulocyte count, peripheral smear, haptoglobin, LDH, direct and indirect bilirubin in addition to DAT, urine hemoglobin or hemosiderin, and bone marrow aspirate/biopsy. Usually, these tests show features of hemolysis and the bone marrow aspirate, and biopsy may show hypo-, normo-, or hypercellularity. In suspected patients with thrombosis, a Doppler scan is recommended. The current gold standard test for the confirmation or exclusion of PNH is a two lineage flow cytometry. The diagnosis is made when a positive flow cytometry is found in the context of clinical features that are suggestive of the condition. The clone size is best estimated based on the larger the white cells clones (monocytes and granulocytes), and in our case, it was greater than 99% on analysis of neutrophils and monocytes. This is also important in prognostication of these patients as those with small clones have good prognosis [16]. The International PNH Interest Group classifies PNH into three main classes: the classical PNH (consists of hemolytic and thrombotic patients); subclinical PNH (small PNH clones without clinical or laboratory evidence of hemolysis or thrombosis); and PNH secondary to other bone marrow disorders (e.g., aplastic anemia or myelodysplastic syndrome) [7].

Management approach to patients with PNH includes treatment of anemia, pain from smooth muscle dystonia, and thrombosis. Anticomplement therapy constitutes an important drug for treating symptoms of PNH. Eculizumab (Soliris), a humanized monoclonal antibody, inhibits the complement-mediated lysis of the blood cells. It reduces the destruction of red blood cells, needs for blood transfusion, and improves the quality of life especially in patients with classical PNH [17]. Eculizumab prevents the breakdown of C5 to C5a and C5b by binding to the complement component of C5. Complement 5a and C5b are required for the formation of the membrane attack complex (MAC). In patients with PNH, RBCs lack CD59 which normally prevent the formation of MAC and the resulting hemolysis. It is indicated in patients with symptoms of disabling fatiguability, patients requiring multiple blood transfusions, pains from smooth muscle spasms, or end-organ dysfunctions [18]. In our case, the PNH is severe and our patient wholly depends on blood transfusions for survival; hence, eculizumab is the therapy of choice. However, its availability issues in addition to the high cost (estimated about 400,000 US dollars per year) limits it uses in Uganda and other developing countries. Due to this fact, we are stuck and currently supporting him with blood transfusions whenever he develops an attack. It should be remembered that the anticomplement therapy is associated with a high risk of Neisseria meningitidis infections, and therefore, immunization and/or prophylactic antibiotic therapy are required [16].

Albeit allogenic hematopoietic stem cell transplantation is considered as a curative therapy for PNH, and it is associated with a number of toxic effects including transplant-related mortality and graft versus host disease [19]. Therefore, it is not commonly used nowadays.

## 4. Conclusion

Our case showed diagnostic and treatment challenges of PNH in a health resource-limited setting.

## Figures and Tables

**Table 1:** Investigations.

Blood tests			
Year	Test name	Results	Lab reference range
2012	CBC		
	(1) WBC	2.2 × 10⁹/L	3.5–10.5 × 10⁹/L
	(2) Hb	3.4 g/dl	13.5–17.5 g/dl
	(3) MCV	116 fl	80–96 fl
	(4) PLT	123 × 10⁹/L	150–450 × 10⁹/L
	Reticulocyte	10.6%	0.5–2.5%
	Peripheral smear	Pancytopenia, dimorphic, normocytic, and normochromic anemia	
	Serum B12 levels	123.2 pmol/L	141–698 pmol/L
	Serum folate levels	45.4 nmol/L	10.4–59 nmol/L
	AST	2.35 ukat/L	0.167–0.667 ukat/L
	Bilirubin total	26 *µ*mol/L	1.7–20.5 *µ*mol/L
	Bilirubin direct	7 *µ*mol/L	5.1 *µ*mol/L
	Antiglobulin test (both direct and indirect)	Negative	
	RDT for malaria	Negative	
	Urine chemistry	Hemoglobin pigments	
	Sickling test	Negative	

2013	CBC		
	(1) WBC	2.6 × 10⁹/L	3.5–10.5 × 10⁹/L
	(2) Hb	5 g/dl	13.5–17.5 g/dl
	(3) MCV	101 fl	80–96 fl
	(4) PLT	130 × 10⁹/L	150–450 × 10⁹/L
	Serum B12 levels	991.6 pmol/L	141–698 pmol/L
	AST	1.2 ukat/L	0.167–0.667 ukat/L
	Peripheral smear	Macrocytosis	
	Bone marrow aspirate and biopsy report	Erythropiosis: hyperplasia with megaloblastic maturation. No granulopoiesis: hyperplastic, left shift with giant metalocytes, and myeloblast <5%	

2014	AST	2.29 ukat/L	0.167–0.667 ukat/L
	Bilirubin total	43.2 *µ*mol/L	1.7–20.5 *µ*mol/L
	Bilirubin direct	5.8 *µ*mol/L	5.1 *µ*mol/L
	LDH	45.38 ukat/L	2.67–7.5 ukat/L
	Antiglobulin test (both direct and indirect)	Negative	
	HBsAg	Nonreactive	
	Hepatitis C antibodies	Nonreactive	
	HIV rapid test	Nonreactive	
	Urine chemistry	Hemoglobin pigments	

2015	Serum homocysteine levels	21 *µ*mol/L	5–16 *µ*mol/L
	Urine methylmalonic acid	0.0 mmol/mol crt	0.0–3.6 mmol/mol crt

2016	Serum B12 levels	1475.6 pmol/L	141–698 pmol/L

2018	CBC		
	(1) WBC	3.4 × 10⁹/L	3.5–10.5 × 10⁹/L
	(2) Hb	3.5 g/dl	13.5–17.5 g/dl
	(3) MCV	111 fl	80–96 fl
	(4) PLT	120 × 10⁹/L	150–450 × 10⁹/L
	Abdominal ultrasound scan	Normal	
	Echocardiography	Dilated chambers of the heart with moderate tricuspid and mitral insufficiency. No features of pulmonary arterial hypertension	

CBC, complete blood count; WBC, white blood count; Hb, hemoglobin; MCV, mean corpuscular volume; PLT, platelets; RDT, rapid diagnostic test; HBsAg, hepatitis B surface antigen; AST, leukocyte aspartate aminotransferase; LDH, lactate dehydrogenase.

**Table 2:** Flow cytometry report.

Blood cells type	GPI-linked antigen type	% blood cells GPI-linked antigen(s) deficiency
Red blood cells	CD59	33.9% (32.4% are type III cells and 1.5% are type II cells)

Neutrophils	FLAER	99.6%
	CD24	
	CD55	
	CD59	

Monocytes	CD14	99%
	FLAER	

CD, cluster of differentiation; GPI, glycosylphosphatidylinositol; FLAER, fluorescent aerolysin. Flow cytometry test was done in South Africa through Lancet Laboratories, Uganda.
